# Supplementary material for: A circuit suppressing retinal drive to the optokinetic system during fast image motion
Source: Nat Commun. 2023 Aug 23;14:5142. doi: 10.1038/s41467-023-40527-z (PMC10447436; doi:10.1038/s41467-023-40527-z)
Supplement: Supplementary file 1 — Supplementary Information [file 41467_2023_40527_MOESM1_ESM.pdf]

A circuit suppressing retinal drive to the optokinetic system during fast image motion.

Adam Mani[1], Xinzhu Yang[1], Tiffany A. Zhao[1], Megan L. Leyrer[1], Daniel Schreck[1], David M. Berson[1]*

[1]Department of Neuroscience, Brown University, Providence, RI, USA.

*Corresponding Author: David M. Berson - david_berson@brown.edu

Supplementary Information

Supplementary Note 1.

Targeting of ON DSGCs for single-cell recording.

We used two-photon illumination to target GFP expressing ON DSGCs in the mouse line HoxD10-GFP, in which all ON DSGCs subtypes and a single subtype of ON-OFF DSGCs express GFP[1]; or the line PCdh9-Cre, in which Cre is expressed by ventral-motion-preferring ON DSGCs[2]. The latter was combined with a Cre-dependent GFP reporter carried by an adeno-associated virus. Alternatively, ON DSGCs were targeted by searching among ganglion cells for their characteristic spiking response to a spot of light projected onto their receptive field center, more sustained and sluggish than for most other RGC types, with a modest onset firing rate[3] (Supplementary Fig. 1a). The identity of the cells was further confirmed by their direction selectivity in response to a full-field grating drifting in different directions (Supplementary Fig. 1b), and by post-recording imaging of their characteristic

dendritic morphology (Supplementary Fig. 1c). ON DSGCs are bistratified, with typically one of the largest dendritic fields in the ON layer among RGCs (dendritic diameter 315 ± 11 µm (n=11), in agreement with[1]), and only a handful of short branches in the OFF layer (Supplementary Fig. 1c). The methods of ON DSGC identification described above did not detect heterogeneity in ON DSGCs that would differentiate subpopulations of cells[4,5] (beyond different preferred directions), although the half maximal response speeds of ON DSGCs covered a relatively broad range (Fig. 1d, blue curve).

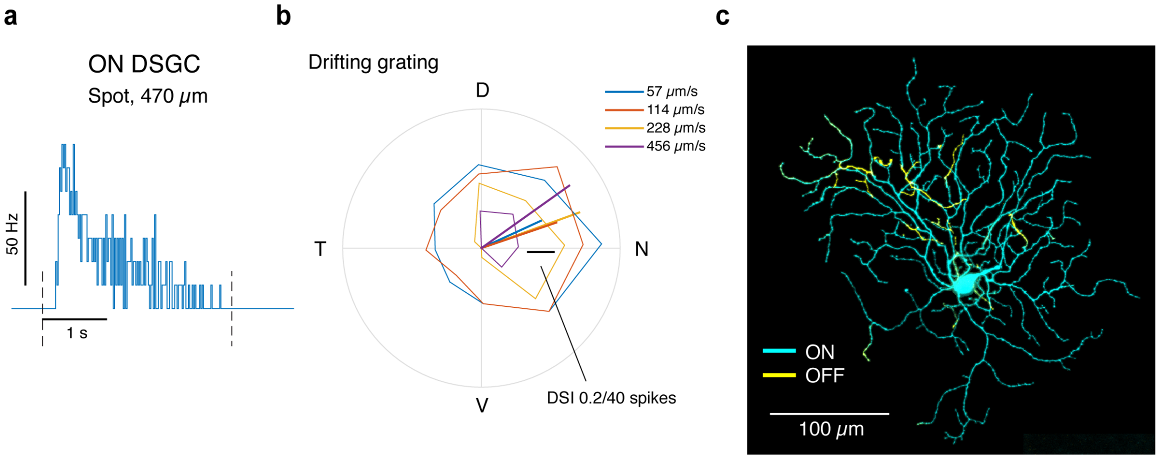

Supplementary Figure 1. Characteristics of ON DSGCs. (a) Characteristic response of an ON DSGC to a bright spot in the center of its receptive field. Mean over 3 repeated trials. Bin Size, 20 ms. (b) Directional tuning of the responses of a single ON DSGC to full-field drifting gratings, for different grating speed. Normalized spike counts, mean over 3 repeats at each speed. Radial colored lines show the preferred directions (angle) and the direction selectivity index (DSI; length) for each speed. (c) Dendritic morphology of an ON DSGC. Cyan, dendrites in the ON layer of the inner plexiform layer. Yellow, dendrites in the OFF layer.

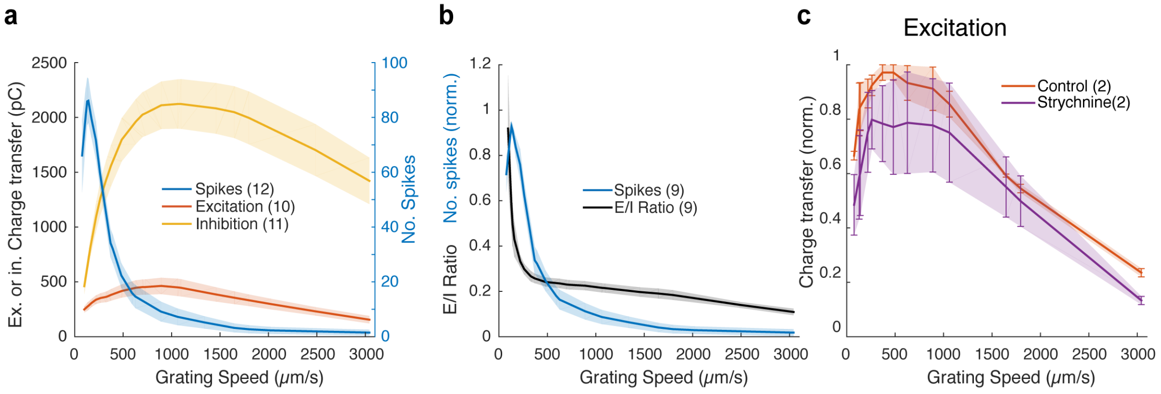

Supplementary Figure 2. Mechanism of ON DSGC suppression at high speeds. (a) The population data from Fig. 1a, non-normalized. Excitation and inhibition in response to full-field drifting gratings are expressed as charge transfer (left vertical axis) and firing – as the total numbers of spikes (right vertical axis). (b) Excitation to Inhibition ratio vs. grating speed, averaged over cells (n=9, black). Normalized firing in the same cells is shown for comparison (blue). (c) Excitation vs. speed did not increase following the blocking of glycine receptors (n = 2 cells), showing that the increase in spiking under this condition is due to reduced inhibition (see Fig. 1g, h). All curves, error bars and shadings in the figure represent the mean ± SEM over cells. Source data are provided as a Source Data file.

Supplementary Note 2.

VGluT3 connectomics: technical approach and additional data.

We mined a publically available serial electron microscopic (SBEM) dataset of the adult mouse inner plexiform layer spanning >200 μm (volume 'k0725'[6]). We reconstructed large numbers of small-field, highly branched amacrine cells known or inferred to be glycinergic[7,8]. We searched for synaptic contacts from such cells onto ON DSGCs, and focused our analysis mainly on a single previously reconstructed presumed ON DSGC (Fig. 2, main text). Numerous anatomical features confirm the identity of this cell, as an ON DSGC, as originally reported[9] (Fig. 2). Its dendrites narrowly costratify with the processes of starburst amacrine cells (SACs), with nearly the entire arbor within the ON SAC plexus (Fig. 2b-e). There, they cofasciculate with SAC processes (Fig. 2a) and receive numerous wrap-around synaptic contacts[10] from SAC varicosities (Fig. 2f, g). The presynaptic SAC processes exhibit the asymmetric connectivity that confers direction selectivity upon DSGCs[9–12]. The minor OFF branches, a common feature of ON DSGC arbors in mice[1], receive some OFF SAC contacts. Presumptive ON-OFF DSGCs in the volume shared many of these attributes but had smaller dendritic arbors, higher branching density, and a larger fraction of their arbor in the OFF SAC plexus.

To identify possible sources of glycinergic amacrine cell inhibition to this ON DSGC, we first marked all the non-ribbon synaptic contacts we could find (n=431; Fig. 2f-i). For each contact, we reconstructed enough of the presynaptic process to sort it into one of three groups (Fig. 2a-e): SACs; wide-field amacrine cells; or candidate

glycinergic neurons. SACs were recognizable from their narrow stratification within the ON or OFF SAC plexus, thin straight connecting dendrites, and large wrap-around varicosities targeting mainly other SACs and DSGCs. Wide-field cells had sparser branching and fewer varicosities and were not strictly confined to the SAC plexuses. Candidate glycinergic neurons were much more highly branched, with arbors that extended well outside the SAC plexuses.

SAC inputs accounted for fully 83% of the non-ribbon contacts (n=340; 317 ON and 23 OFF). At least by this measure, GABAergic SAC input dominates the inhibition of ON DSGCs. Candidate glycinergic inputs (n=41) were the next most common, comprising 10% of non-ribbon inputs, with wide-field inputs nearly as common at 7% of all input (n=27). A small minority of inputs (n=23; 5%) could not be identified because too small a fraction of the arbor could be reconstructed, and were excluded from the sample.

We extensively reconstructed all of the presumptive glycinergic neurons identified by our initial screen. To our surprise, nearly all of them appeared to be VGluT3 amacrine cells, as documented below (n=38 synapses from 16 cells). Two were synapses from Type H18 amacrine cells[13,14] and one was from an unidentified medium-field type. We detected no synaptic inputs from any of the many other types of small-field amacrine cell types, even after examining a large sample of such

cells reconstructed in another study[14]. We conclude that VGluT3 amacrine cells are by far the best candidate glycinergic cell type shaping the slow-speed tuning of ON DSGCs.

Multiple structural observations confirm these as VGluT3 amacrine cells (Supplementary Fig. 3). First, their stratification is appropriate (Supplementary Fig. 3e, f, j). They arborize most heavily in the middle of the inner plexiform layer (IPL), between the ON and OFF SAC plexuses, but extend sparse processes into and even a bit beyond those plexuses. Their cell bodies lie in the inner nuclear layer, are of appropriate size, and are distributed with spacing that suggests a regular mosaic (Fig. 3a). Their dendritic-field diameters (106 ± 7 μm, n = 10 cells) are in line with earlier data[15,16] and overlap extensively with a coverage factor of at least 5 (Supplementary Fig. 3a, inset).

Input and output patterns matched previous reports on VGluT3 cells, and were very consistent across the population of reconstructed cells (Supplementary Fig. 3i, Supplementary Table 1), confirming other evidence that they belong to a single type. In keeping with functional evidence for input from both the ON and OFF pathways to VGluT3 cells, our reconstructed cells receive ribbon synapses from diverse ON and OFF bipolar cells. The complement of bipolar inputs largely reflects their dendritic stratification (Supplementary Fig. 3g, i) with most input from OFF types 3a, 3b, and 4 and ON types 5o, 5i, 5t, and little from bipolar types stratifying at the IPL margins (OFF types 1,2 and GluMI[17]), and ON types 6, 7, 8 and 9. Their outputs

include all the ganglion-cell targets so far identified in physiological studies (Supplementary Table 1). These included W3 cells[18,19], OFF transient alpha cells[20], Suppressed-by-Contrast cells of several types[20,21] including the ON Delayed RGC[22] (see Discussion), both ON and ON-OFF DSGCs[18,19], and M1 ipRGCs[23] (For RGC type nomenclature see [3] and accompanying online database). However, our SBEM analysis suggests that their outputs are much more diverse than previously appreciated, with contacts to the great majority of known RGC types[3], including OFF sustained alpha, Jam-B[24], ON alpha, F-mini-ON[25], F-mini-OFF[25], OFF transient medium RF, and diverse varieties of smaller field RGCs stratifying between the ChAT bands[26]. Two ipRGC types notably not targeted by VGluT3 are the M2[27] and M5 (PixON)[28,29] types, which stratify largely outside the VGluT3 plexus. VGluT3 cells also synapsed upon diverse types of amacrine cells, though only rarely onto other VGluT3 cells or SACs. We have not reconstructed most amacrine-cell targets, though WF cells stratifying between the SAC plexuses were among the most common recipients[30]. VGluT3 outputs, like their ribbon inputs, were distributed most heavily within and between the SAC plexuses (Supplementary Fig. 3h), though they were also sparsely present in the distal IPL. Some of these are somato-dendritic synapses onto M1 ipRGCs. One unexpected feature of the VGluT3 cells in this volume was a consistent upward (roughly dorsal) displacement of the dendritic arbor relative to the soma (Supplementary Fig. 3b-d).

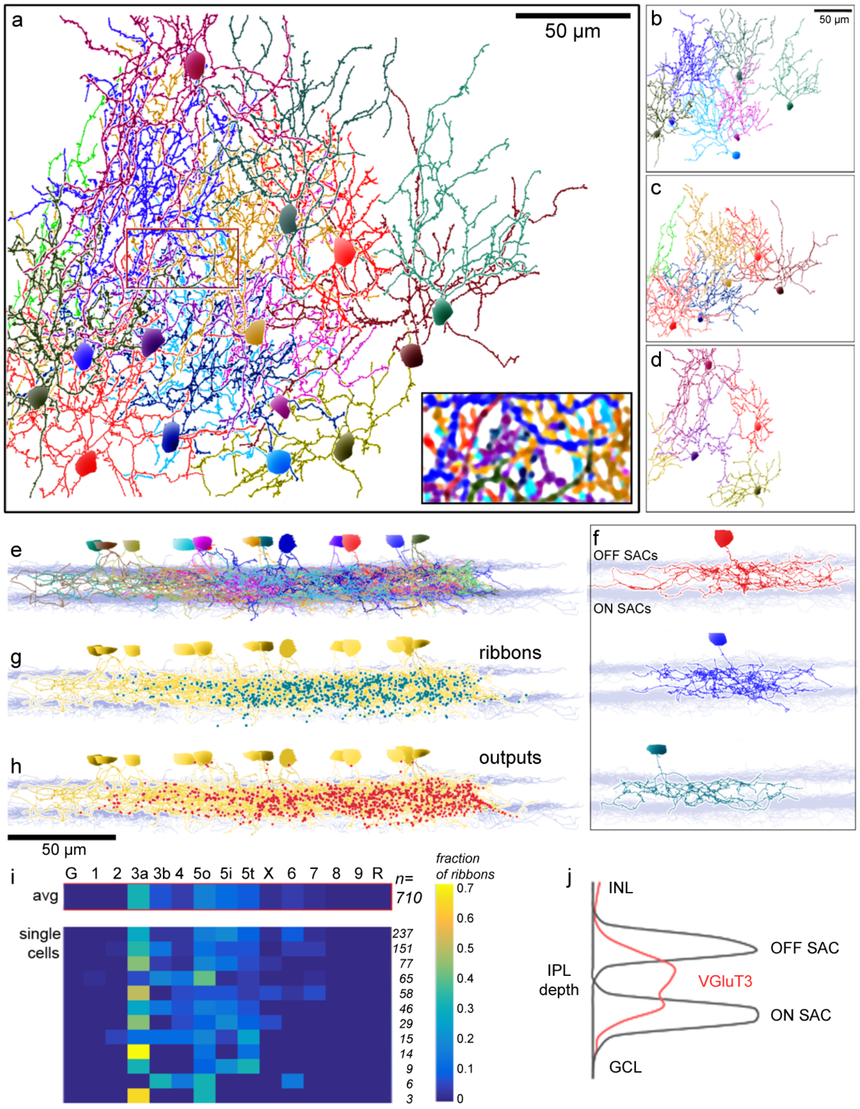

Supplementary Figure 3. Morphology, input and output of VGluT3 amacrine cells. (a-d). Dendritic arbors and mosaic of VGluT3 amacrine cells reconstructed in the k0725 SBEM volume as seen in top view (*en face*). Enlarged view (inset) shows that local regions contain dendrites of at least 5 VGluT3 cells. (b-d): Same dendritic profiles as in A but divided arbitrarily into three groups to provide a clearer view of single arbors. (e,f) Side (vertical) views of the stratification of reconstructed VGluT3 cells within the IPL, shown together (e) and, for 3 cells, individually (f). (g, h) Side view of the location in depth of ribbon inputs (f) and synaptic outputs (g) of VGluT3 cells. Gray bands in e-h mark the ON and OFF SAC plexuses; VGluT3 cells appear gold in g and h. (i) Heatmap plot of the fraction of all ribbon synaptic inputs derived from each bipolar type, identified at the top (G – GluMI, X – XBC, R – rod bipolar cell). Bar at top shows pooled data for all identified ribbon contacts (n=710). Lower panel illustrates the consistency of this pattern across individual cells, shown one per row in descending order of the number of identified ribbons. (j) Stratification profile of reconstructed VGluT3 processes (red; n=17 cells) in relation to those of ON and OFF SACs (black). Data are normalized to the ON SAC plexus.

Supplementary Note 3.

A VGluT3-Cre mouse line for specific manipulation of VGluT3 amacrine cells.

In order to manipulate VGluT3 cells in a specific manner, we studied Cre-expression in the mouse line VGluT3-IRES2-cre-D that to our knowledge had not been previously used in retinal studies. To this end we crossed the VGluT3-Cre mouse with a tdTomato reporter mouse (Ai14). In retinas of these mice, nearly every VGluT3-immunopositive neuron expressed tdTomato (98.2 ± 0.6%; 9 images, 2 retinas; Supplementary Fig. 4a). VGluT3-cell dendrites formed a dense plexus concentrated between the ON and OFF SAC plexuses, as revealed by anti-ChAT immunofluorescence (Supplementary Fig. 4e). The only other retinal cells brightly labeled with tdTomato were Müller glia, though we occasionally encountered labeled RGCs (~20/mm^2) and wide-field amacrine cells with somas in the GCL or INL and straight, sparsely branches processes in the IPL (~2 processes in a 200x200 μm field of view).

To gain optogenetic and chemogenetic access to VGluT3 cells, we crossed the VGluT3-Cre mouse with lines expressing channelrhodopsin (Ai32), or the hM4Di receptor (DREADD), respectively. To verify expression of the reporters in VGluT3 cells in the crossed mice, we used the YFP expressed along with ChR2 in the VGluT3 xAi32 mouse, and an antibody against HA tag was used in VGluT3 xR26 (DREADD) (Supplementary Fig. 4b, c). VGluT3 were labeled independently using anti-VGluT3 antibodies, and SACs were labeled using anti-ChAT. In VGluT3-Ai32 mice, YFP

expression was usually seen during recordings using 2-photon or single-photon illumination.

To obtain information on the morphology of the Cre expressing cells in the VGluT3-Cre line, we injected adeno-associated viruses (AAV) expressing GFP or YFP in a Cre dependent manner into the eyes of VGluT3-Cre mice. This often resulted in labeling that was sparse enough for discerning the dendrites of individual cells (Supplementary Fig. 4d). The morphology of the labeled cells matched that of VGluT3 ACs ([15,16,31] and SBEM data in this study). In addition, the GFP virus resulted in more non-VGluT3 cells expressing GFP than the crossed mouse line VGluT3 xAi14, possibly due to leakiness of the promoter carried by the AAV.

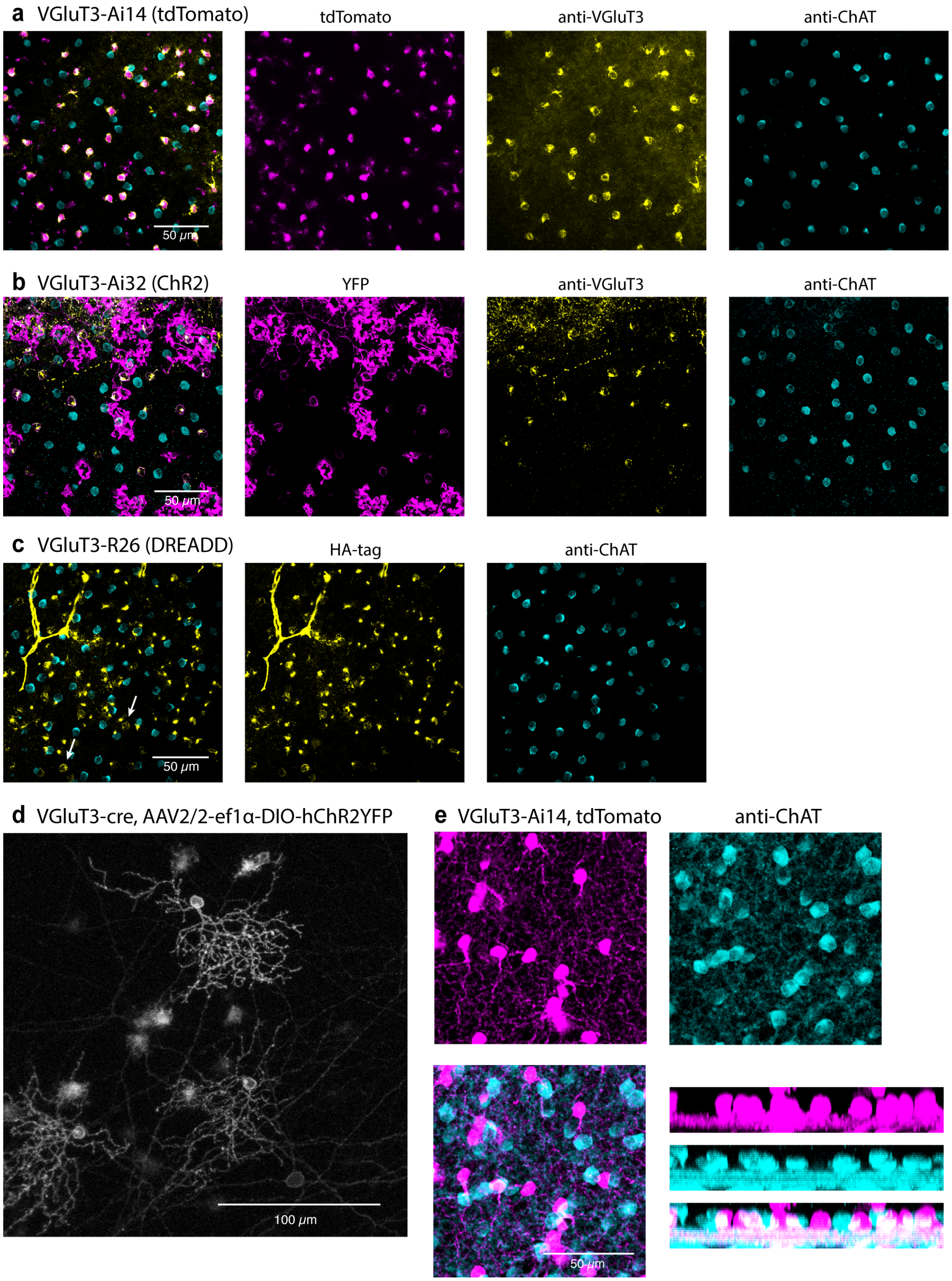

Supplementary Figure 4. Characterization of the VGluT3-Cre mouse and its hybrids with reporter mice. Confocal stacks of VGluT3 and ChAT immunostaining together with (a) tdTomato expressed in VGluT3xAi14. (b) YFP expressed in VGluT3xAi32 (ChR2-YFP). (c) HA tag expressed in VGluT3xR26 (hM4Di-HA). (d) Sparsely labeled VGluT3 cells in a VGluT3-Cre mouse injected with an AAV carrying ChR2-YFP. (e) Top and side views of VGluT3xAi14 at high magnification, showing a VGluT3 dendrite plexus between the two ChAT bands.

Supplementary Note 4.

ON DSGC inhibition in response to optogenetic pulse trains.

While recording inhibitory currents in ON DSGCs, we delivered optogenetic pulse trains to mimic the temporal modulation of VGluT3 cells during grating motion (Supplementary Fig. 5c). Evoked inhibitory currents persisted in postsynaptic ON DSGCs throughout a 5 s train over the full range of frequencies tested (1-8 Hz, corresponding to grating speeds of roughly 400 - 3000 μm/s for experiments shown in Fig. 1). At higher stimulus frequencies, IPSCs summed temporally to produce a continuous inhibitory conductance (Supplementary Fig. 5c, bottom traces), mirroring the conductances evoked during rapid grating motion (Fig. 1b, yellow traces). This experiment demonstrates that inhibitory synapses from VGluT3 cells onto ON DGSCs can produce postsynaptic currents with kinetics compatible with their proposed role in vetoing responses to fast motion.

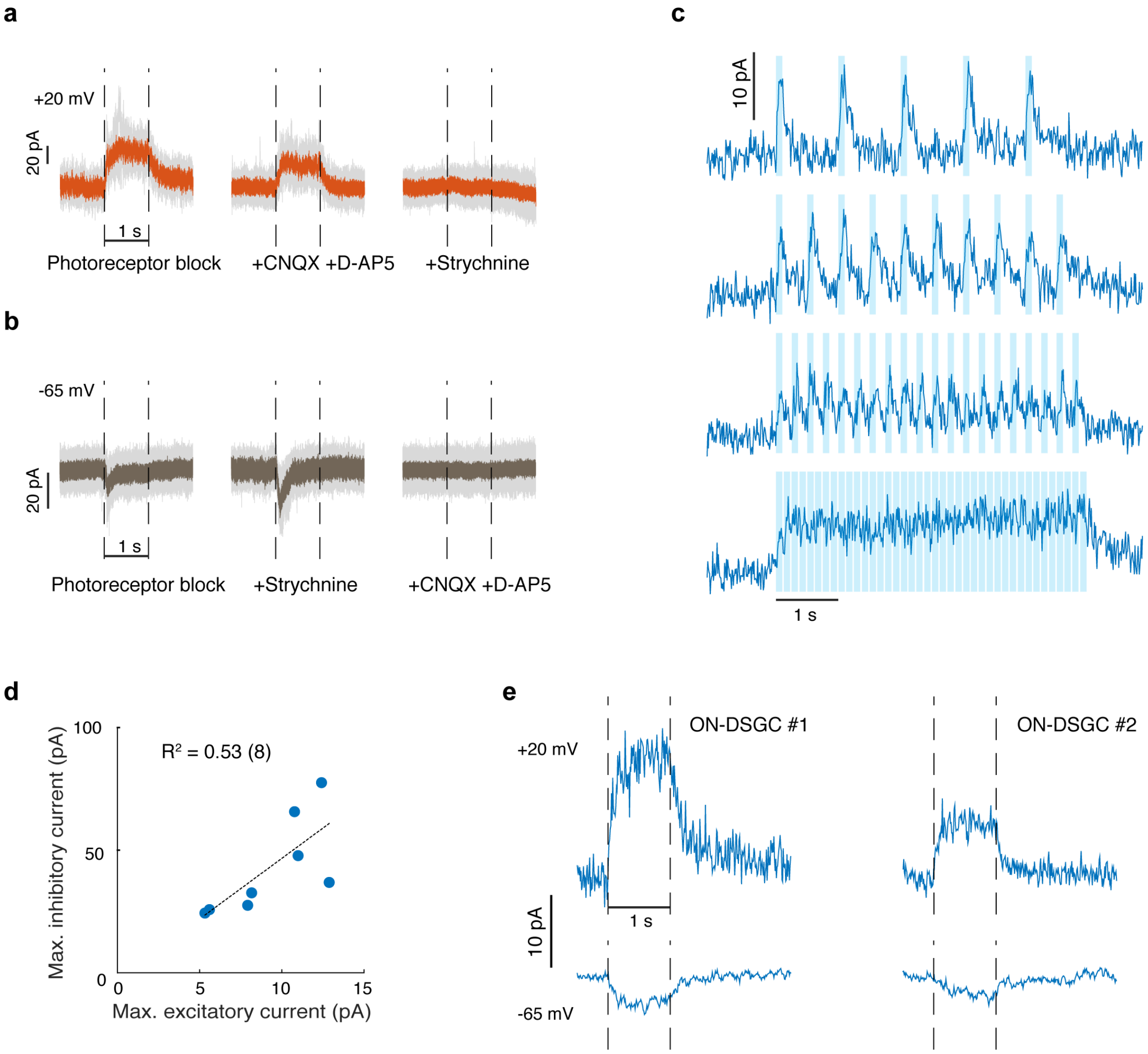

Supplementary Figure 5. Optogenetics supplementary data. (a) Raw inhibitory current trace (V_{hold}: +20 mV) in an ON DSGC in response to optogenetic stimulation. The same data following smoothing is shown in Fig. 3a, left panel. Red, mean over 3-5 repeated trials that are shown in gray. (b) Raw excitatory current trace (V_{hold}: -65 mV) induced in an ON DSGC by optogenetic stimulation. The smoothed version is shown in Fig. 3g. Brown, mean over 3-5 repeated trials shown in gray. (c) Inhibitory currents (V_{hold}: +20 mV) in an ON DSGCs in response to optogenetic pulse trains of frequencies 1, 2, 4, 8 Hz. Pulse width: 100 ms. (d) Maximal inhibitory and excitatory currents induced in the same ON-DSGCs by optogenetic depolarization of VGluT3 cells (n = 8 cells). Dashed line and R^2 value are the results of a linear regression. (e) Example traces of excitatory and inhibitory currents induced optogenetically in the same ON-DSGCs (two different cells). Traces in c and e are averaged over 3-5 repeated trials, and smoothed using a running average in a 10 ms window. Source data are provided as a Source Data file.

Supplementary Note 5.

Controls for optogenetic studies.

Optogenetically induced currents typically exhibited rundown, with smaller responses after repeated stimulation. In a pharmacological experiment, such rundown could masquerade as a drug effect. To assess the extent of the rundown effect, we conducted sham experiments (Supplementary Fig. 6a). Using the standard photoreceptor block but no subsequent drug application, we measured the optogenetically evoked currents at intervals similar to those in an actual pharmacological experiment. Over the time required to complete a real drug trial with two steps of drug application, the peak current dropped by 38 ± 2% (2 cells), but was not eliminated. We conclude that while rundown does contribute to the decreases of the current between steps in these experiments, the pharmacological effects we report are real.

In VGluT3 x Ai32 mice, we observed the expression of the YFP reporter in Müller glia (Supplementary Fig. 4b). It was therefore important to demonstrate that optogenetic depolarization of Müller glia does not contribute the induced currents in ON DSGCs, especially in light of recent evidence for electrical coupling between Müller glia and amacrine cells[32]. To this end, we crossed a Müller-specific Cre mouse (GLAST-creER)[33] with the Ai32 mouse. In these animals, ON DSGCs exhibited neither excitatory nor inhibitory currents during photostimulation of the Müller cells (Supplementary Fig. 6b).

The currents induced optogenetically in VGluT3 x Ai32 mice are due to the ChR2 transgene, and not to other light-dependent mechanisms. In control recordings of ON DSGCs in HoxD10 mice without viral or genetic expression of the channelrhodopsin, the optogenetic stimulus evoked no currents (Supplementary Fig. 6c). This implies that the pharmacological cocktail used in optogenetic experiments to block rod and cone influences on ON DSGCs was largely effective. Rarely, an optogenetic stimulus appeared to trigger large current bursts; these may have resulted from incomplete blocking of rod/cone networks in some cells. Those were mostly inconsistent over trials and had a longer latency compared to persistent optogenetic responses, and were excluded from the data. A persistent ACET-resistant current was recorded in a minority of ON DSGCs, appearing as an OFF response upon termination of the LED stimulation (Supplementary Fig. 6c). Incomplete blockade of light responses tended to be more pronounced in the presence of strychnine.

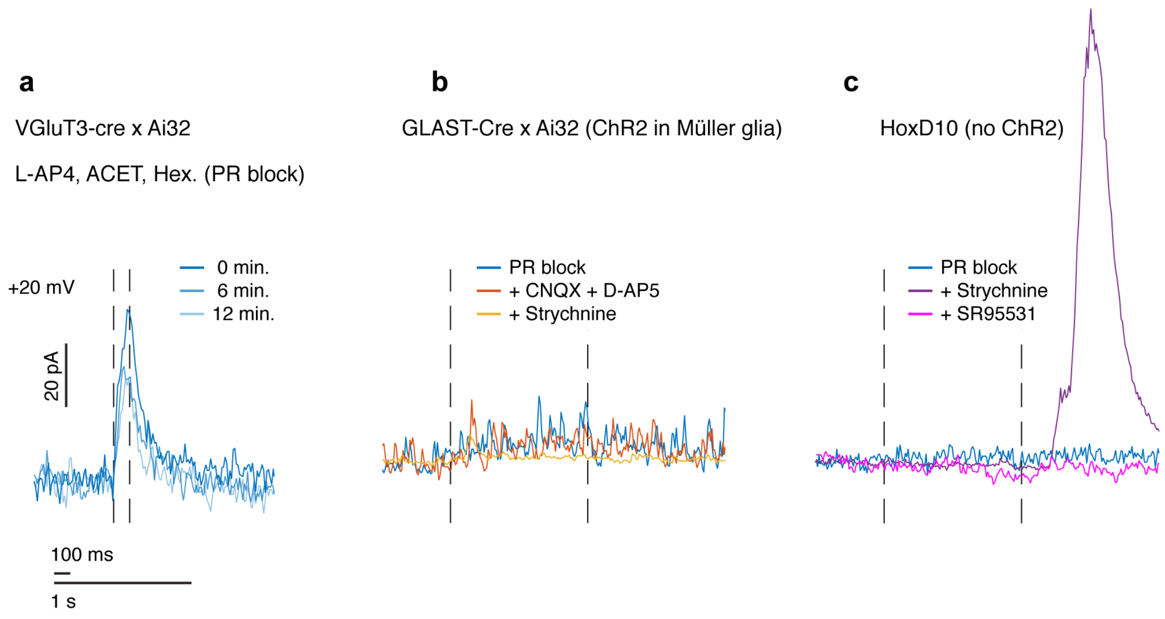

Supplementary Figure 6. Optogenetics control experiments. (a) Sham three steps experiment with photo-transduction and acetylcholine block ('PRblock') but without additional blockers added, to test rundown of the optogenetically induced inhibitory current in an ON DSGC. (b) ON DSGC inhibition in a mouse expressing ChR in Müller glia, in response to the LED stimulus, with PR block followed by the addition of glutamate transmission block and strychnine. (c) ON DSGC inhibition in a mouse expressing no ChR, with PR block, followed by the addition of strychnine and SR95531. In a-c, the holding voltage is the excitation reversal potential, and the LED stimulus time is between the dashed lines. Traces in figure are averages over 3-5 repeated trials, and smoothed using a running average in a 10 ms window.

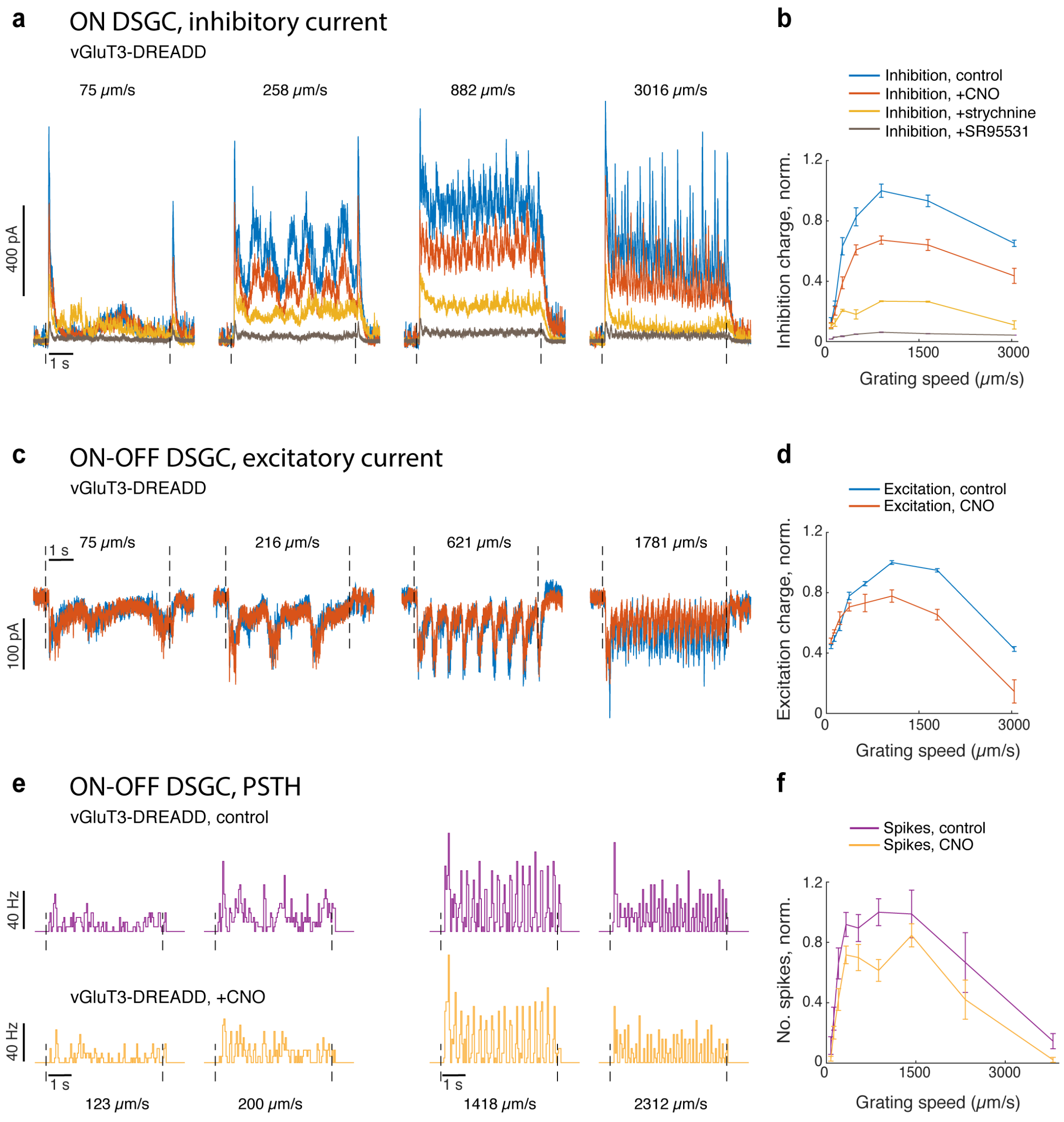

Supplementary Figure 7. (a) Inhibitory current traces in an ON DSGC in response to gratings of different speeds, in VGluT3-DREADD mouse. Inhibition was reduced by application of the DREADD ligand CNO but was further reduced by strychnine. The remainder was removed by the $GABA_A$ receptor blocker SR95531. Grating speeds are denoted above the traces. (b) Speed response curves for the same cell as the traces in a. (c) Excitatory current in an ON-OFF DSGC in a VGluT3-DREADD mouse is reduced by CNO. (d) Speed-response curves for the same cell as the traces in c. (e) Spike responses (PSTH) to the grating in an ON-OFF DSGC were reduced by CNO. (f) The speed response curve for the same data. All curves, current traces and PSTH in the figure are averaged over 3 repeated trials. Error bars represent SEM over trials. Source data are provided as a Source Data file.

Supplementary Note 6.

Origin of fast-motion inhibition in ON DSGCs not blocked by DREADD.

Strychnine reduced inhibition in ON-DSGCs across speeds more than the chemogenetic suppression of VGluT3 cells did (Fig. 1d, Fig. 4d, e, Supplementary Fig. 7a, b). The chemogenetic manipulation therefore only partially suppressed glycine release onto ON DSGCs.

Overall, DREADD-mediated suppression of VGluT3 cells eliminated only about half of the inhibition induced in ON DSGCs by fast motion (Fig 5a, b). The remaining inhibitory current might be supplied by a different type of amacrine cell, but it could also stem from incomplete suppression of VGluT3 cells. Application of the DREADD ligand CNO scaled down the inhibitory currents while preserving the kinetics (Supplementary Fig. 8a), and the scaling factor was nearly constant across different grating speeds (Supplementary Fig. 8b). This is most easily explained by incomplete suppression of VGluT3 cells. If a different amacrine type is responsible, it must exhibit temporal kinetics and ON vs. OFF sensitivity closely matched to that of VGluT3 cells.

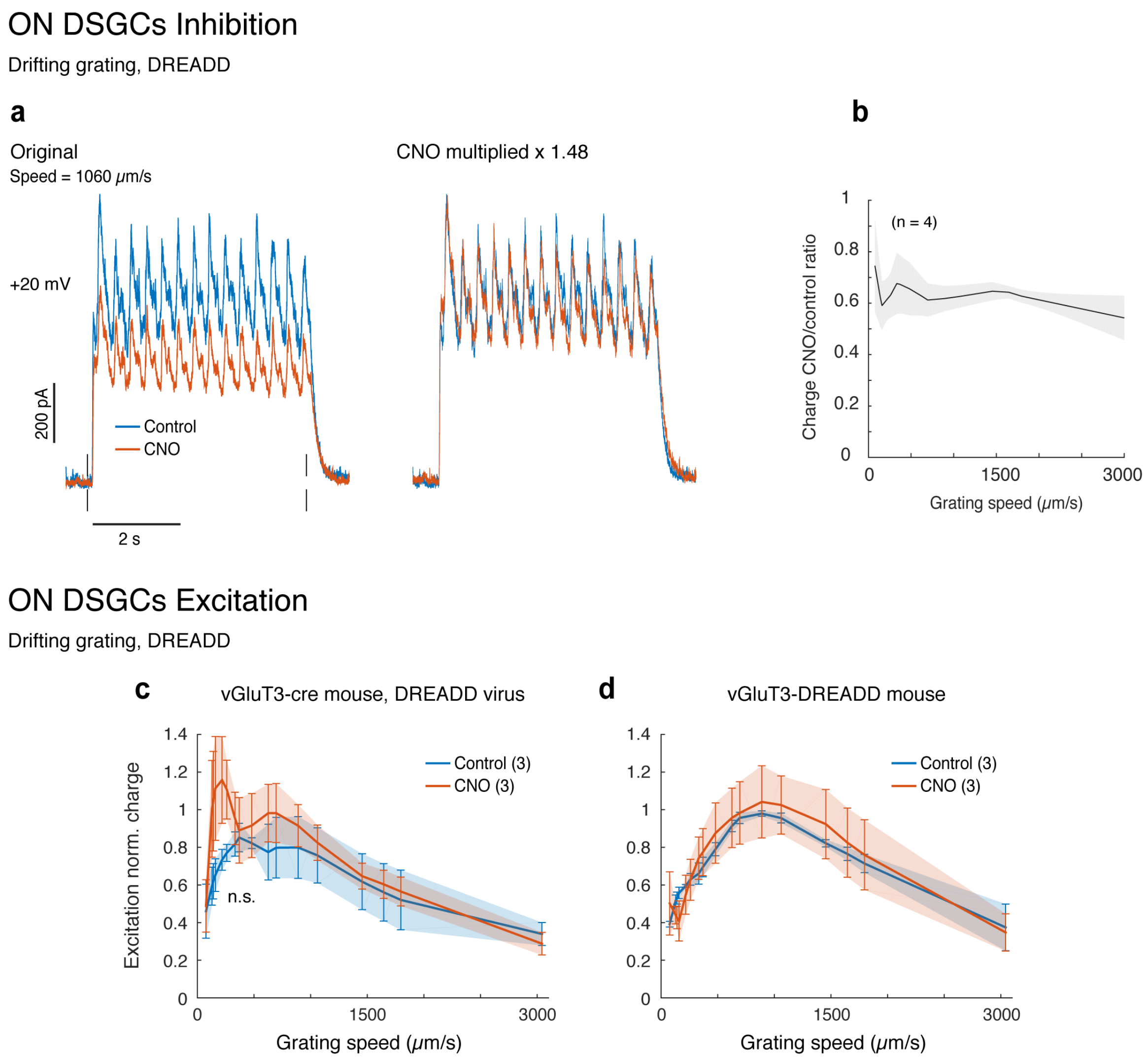

Supplementary Figure 8. Synaptic currents in ON DSGCs with DREADD VGluT3 Suppression. (a) Scaling down of inhibition: inhibitory current (mean over 3 repeated trials) in an ON DSGC before and after the application of CNO (left), and the same currents with the CNO trace multiplied x 1.48, to show high similarity of the curves. The stimulus was a grating drifting in the preferred direction. (b) The ratio of charge transfer before and after CNO, for different grating speeds. Mean ± SEM, 4 cells. (c, d) Application of CNO (red trace) did not significantly affect excitatory currents evoked by moving gratings in ON DSGCs under control conditions (blue), with DREADD expressed either by a Cre-dependent virus (c) or through a genetic cross (d). Curves, error bars and shadings in c, d represent mean ± SEM over cells. The difference between the control and DREADD conditions was insignificant for curve maxima or at all data points, in a paired two-sided t-test (3 cells for virus, 3 for cross). Source data are provided as a Source Data file.

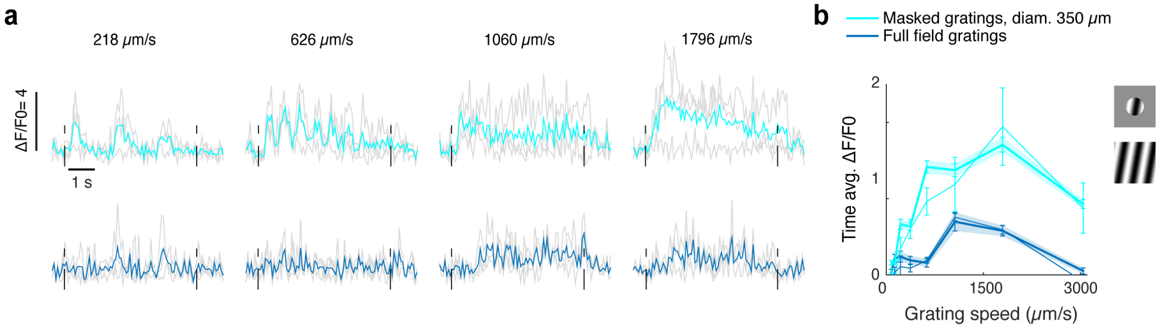

Supplementary Figure 9. VGluT3 calcium responses to full-field vs. masked gratings. (a) Average fluorescence traces (3 repeated trials) from example ROIs for full-field grating (dark blue), and a grating limited to a circular mask (350 μm; light blue) centered on the same FOV. Gray lines are individual trials. Grating speeds are shown over the traces. (b) Mean ± SEM of 70 ROIs (masked gratings) or 12 ROIs (full-field gratings) in the FOV are shown as thick lines and shading, while curves for the specific ROIs of traces in a are shown as thin lines, and represent mean ± SEM over 3 repeated trials. Colors are as in a. Stimulus schematics are shown on the right. Source data are provided as a Source Data file.

Supplementary Note 7.

Spots and gratings area-response functions in VGluT3 dendrites.

We compared the receptive field size and surround suppression, when measured with gratings vs. spots (Fig. 5g, h, Supplementary Fig. 10a, b). The surround stimulation using spots was a simple step in luminance whereas the grating stimulus introduced local contrast as well as motion stimuli without a change in integrated luminance. We thus tested new sets of spots and gratings, with overall contrast in the two stimuli equal and positive (Supplementary Fig. 10c, d, see Methods). The area-response functions for spots resembled those seen previously, although the lower contrast weakened the responses. The surround suppression for masked gratings, however, was stronger than for zero mean gratings. Although the optimal size of the stimulus and the suppression at the maximal size were no longer different between spots and gratings, the shape of the response functions were still markedly different, with the response stronger for gratings at larger sizes (Supplementary Fig. 10d). In summary, the weaker surround suppression in the case of gratings vs. spots, is both due both to a smaller change in contrast as well as to the continuous motion.

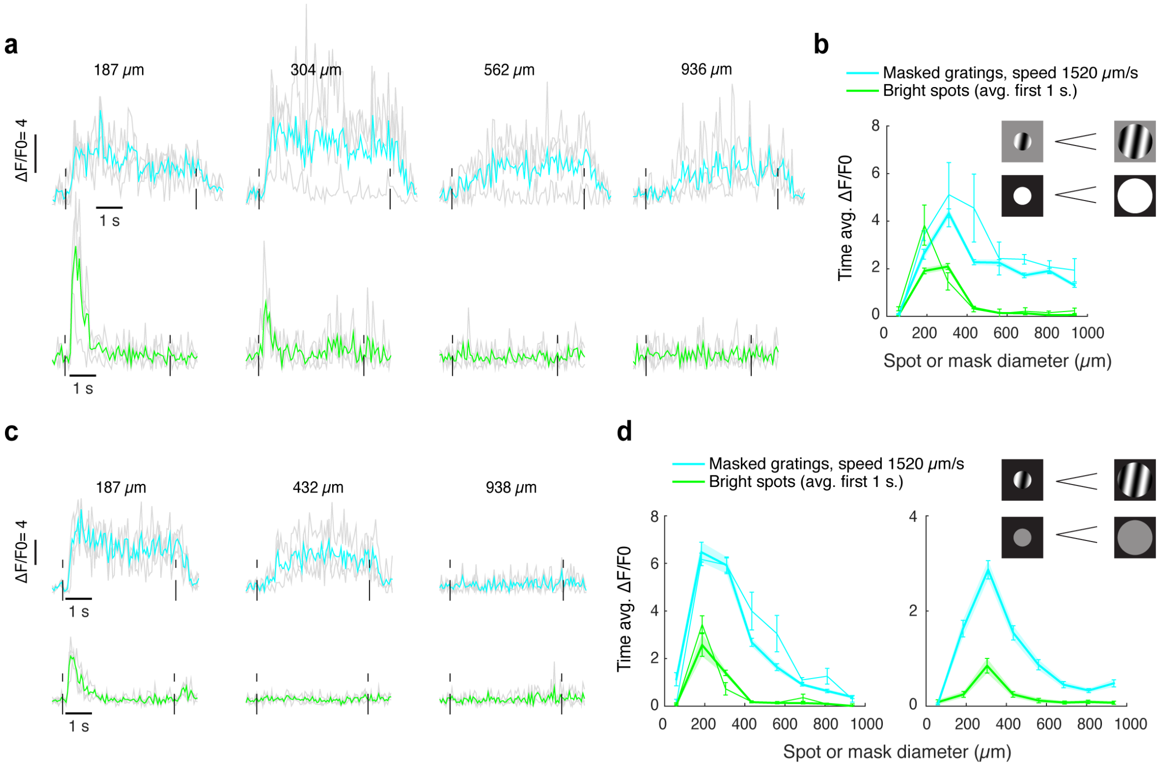

Supplementary Figure 10. Gratings and spots area response functions in VGluT3 dendrites. (a) Traces from specific ROIs for area response functions, measured with bright spots (green) and masked gratings (light blue). Mean over 3 repeated trials, individual trials shown in gray. For gratings, the size of the mask was varied while the grating and its speed (1520μm/s) remained the same. Mask or spot diameters appear over the traces. (b) Thick lines and shading are response mean ± SEM over ROIs in the same FOV (51 ROIs for spots, 73 for gratings), and the thin lines are the specific ROIs from which the traces in a were taken (mean ± SEM over trials). Colors are as in a. Stimulus schematics are on the right. (c) Same as in a, but here the two stimuli were contrast equated (see difference in stimulus schematics between b and d). Spots now had the same average contrast as the gratings (50% relative to a), while the gratings were presented from dark rather than gray. (d) mean ± SEM of response over ROIs in two different FOVs (left: 39 ROIs for gratings, 28 for spots, and right: 85 ROIs for gratings, 28 for spots). Thin lines are mean ± SEM over the repeated trials in the ROIs of the traces in c. Colors are as in c. Source data are provided as a Source Data file.

Supplementary Note 8.

Directional independence of ON DSGC glycinergic inhibition.

We expected that the orientation or direction selectivity found in VGluT3 dendrites would be reflected in the feedforward glycinergic inhibition to ON DSGCs (Supplementary Fig. 11a). To our surprise, however, we did not detect a dependence on the grating's direction of motion. At the optimal grating speed for evoking inhibition (1220 μm/s), the inhibitory current was neither OS nor DS (OSI = 0.06 ± 0.01, DSI = 0.07 ± 0.01, n = 6 cells, Supplementary Fig. 11a). When glycine receptors were blocked with strychnine, the remaining GABAergic inhibition revealed a stronger bias, as expected from the dominant SAC input. The glycinergic contribution to the current was assessed by subtracting the charge transfer under strychnine from that of the control condition. At this high speed, glycinergic current was dominant and relatively insensitive to stimulus direction (n = 2 cells). Glycinergic inhibition was also directionally isotropic at a slow speeds (Supplementary Fig. 11b). As another attempt to isolate glycinergic inhibition and test for OS/DS, we blocked GABA receptors using SR95531 (Supplementary Fig. 11c). That, however, led not to a decrease but rather an increase in the current, potentially due to a release from dis-inhibition upstream, which may have removed OS/DS in the VGluT3 dendrites. Indeed, the same test on Ca^{2+} responses in VGluT3 dendrites resulted in a dramatic increase in the signal and a reduction of orientation selectivity (n = 2 FOVs; Supplementary Fig. 11d), consistently with its role in providing glycinergic inhibition to ON DSGCs.

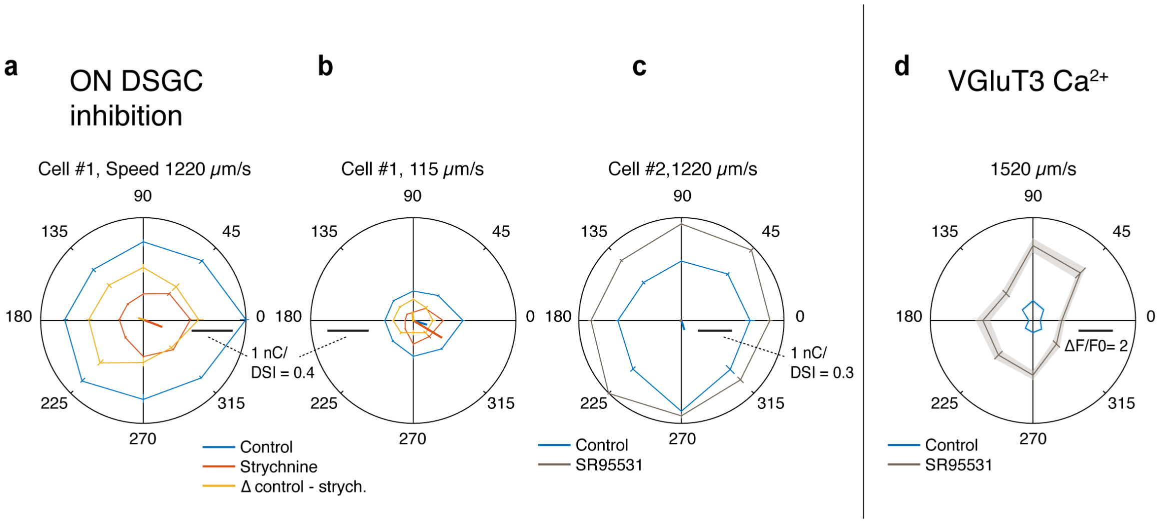

Supplementary Figure 11. Directional independence of ON DSGC inhibition (a) Inhibition in ON DSGCs (whole cell voltage clamp, V_{hold}: +20 mV), in response to full-field gratings moving in different directions at the speed denoted above. Inhibitory charge before (blue) and after strychnine addition (red) are shown, as well as the difference between the two, attributable to glycine (yellow). Radial colored lines represent the preferred direction (angle) and direction selectivity index (length). (b) Same as in a for a slower speed denoted above. a and b were recorded in the same ON DSGC. (c) Inhibitory charge before (blue) and after $GABA_A$ receptors (SR95531) blockade (brown) in an ON DSGC. Curves and error bars in a-c are mean ± SEM over 3 trials. (d) Effect of blocking of $GABA_A$ receptors on the Ca^{2+} signal in VGluT3 dendrites (mean ± SEM in a FOV, 67 ROIs for control, 48 for SR95531) is shown for comparison to panel c (colors are as in c). Source data are provided as a Source Data file.

	TYPES	EyeWire [34]	RGCtypes.org [3]	Helmstaedter et al. [13]	Other reference	Synapses	% of cell class (RGC, AC, BC)
**RGCs**	All types					537	60% of entire sample
	M1	1ws	M1			3	1
	M2(8)	9w	M2			3	1
	M2(9)	9w	M2			0	0
	M4	8w	ON alpha			4	1
	M5	8n/9n?	PixON	12		0	0
	M6	91, 915, 81o	M6			1	0
	ON DS	7id, 7ir, 7iv	ON DS			49	9
	ON-OFF DS	37c,d,r,v	ON-OFF DS	9		86	16
	OIA	6sw				20	4
	OIB	6sn	ON transient small RF			15	3
	ON transient	6t	ON transient			11	2
	LAMBDA(3a4)	5so, 51?	HD/LED	5,10, 11?		12	2
	LAMBDA(5oi)	5so, 51?	HD/LED	5,10, 11?		18	3
	LAMBDA(5t)	5so, 51?	HD/LED	5,10, 11?		6	1
	W3	5ti	UHD	7		63	12
	F-mini ON	63	F-mini ON	6		29	5
	OFF trans ALPHA	4ow	OFF trans alpha			49	9
	OMTA	4on	OFF trans medium RF			34	6
	delta	1wt	OFF sustained alpha	3		2	0
	delta-like	2aw, 2i, 2o, 3i,3o	OFF sus., OFF medium sus., OFF hOS	1		3	1
	rho	25		2		4	1
	F-mini OFF	1no, 1ni, 2an,	F-mini OFF			0	0
	JAMB	2w	OFF vOS			1	0
	R	73	ON delayed	8		19	4
	SBC1	81i, 82wi, 82wo	OS			31	6
	SBC2	72, 82n	OS			11	2
	RGC unident.					63	12
**ACs**	All types					337	38% of entire sample
	A17			49	[35]	0	0
	CRH1			55	[36, 37, 38]	1	0
	CRH3			54	[36]	0	0
	ON SAC			51		2	1
	RAC1				[38]	0	0
	SFE			43		8	2
	WF(5i)					2	1
	WF interChAT					25	7
	nNOS1				[36, 40]	2	1
	AII			24		0	0
	DAC					0	0
	H16			16		0	0
	H18			18		0	0
	H19			17/19		0	0
	H21			21		0	0
	H22			22		0	0
	H23 MAC			23	[32]	0	0
	H36			36		0	0
	H45			45		5	1
	H52			52		1	0
	OFF SAC			33		1	0
	SF OFF					0	0
	TH2				[41]	6	2
	VGluT3					1	0
	VIP-RAC2				[39]	7	2
	WF ON ChAT + M4					1	0
	AC OTHER/ unident.					275	82
**BCs**	All types					22	2% of entire sample
	GluMI				[42]	0	0
	1					1	5
	2					0	0
	3a					6	27
	3b					0	0
	4					4	18
	5o					3	14
	5i					0	0
	5t					3	14
	XBC				[3]	0	0
	6					2	9
	7					0	0
	8					0	0
	9					0	0
	RBC					2	9
	unident.					1	5

Supplementary Table 1. Output synapses and postsynaptic cells from VGluT3 cells. n = 896 synapses from 16 VGluT3 cells in the K0725 SBEM dataset, organized according to their postsynaptic cells. Since this sample is not random, numbers and percentages may be biased, for example for ON DSGCs. Cell types where matched with nomenclature in three existing datasets, and/or in previous literature.

Supplementary references

1. Dhande, O. S. *et al.* Genetic dissection of retinal inputs to brainstem nuclei controlling image stabilization. *J. Neurosci.* **33**, 17797–17813 (2013).
2. Lilley, B. N. *et al.* Genetic access to neurons in the accessory optic system reveals a role for Sema6A in midbrain circuitry mediating motion perception. *J. Comp. Neurol.* **527**, 282–296 (2019).
3. Goetz, J. *et al.* Unified classification of mouse retinal ganglion cells using function, morphology, and gene expression. *Cell Rep.* **40**, 111040 (2022).
4. Kanjhan, R. & Sivyer, B. Two types of ON direction-selective ganglion cells in rabbit retina. *Neurosci. Lett.* **483**, 105–109 (2010).
5. Gauvain, G. & Murphy, G. J. Projection-specific characteristics of retinal input to the brain. *J. Neurosci.* **35**, 6575–6583 (2015).
6. Ding, H., Smith, R. G., Poleg-Polsky, A., Diamond, J. S. & Briggman, K. L. Species-specific wiring for direction selectivity in the mammalian retina. *Nature* **535**, 105–110 (2016).
7. Heinze, L., Harvey, R. J., Haverkamp, S. & Wässle, H. Diversity of glycine receptors in the mouse retina: localization of the alpha4 subunit. *J. Comp. Neurol.* **500**, 693–707 (2007).
8. Zhang, C. & McCall, M. A. Receptor targets of amacrine cells. *Vis. Neurosci.* **29**, 11–29 (2012).

9. Matsumoto, A., Briggman, K. L. & Yonehara, K. Spatiotemporally Asymmetric Excitation Supports Mammalian Retinal Motion Sensitivity. *Curr. Biol.* **29**, 3277-3288.e5 (2019).

10. Briggman, K. L., Helmstaedter, M. & Denk, W. Wiring specificity in the direction-selectivity circuit of the retina. *Nature* **471**, 183–190 (2011).

11. Yonehara, K. *et al.* Spatially asymmetric reorganization of inhibition establishes a motion-sensitive circuit. *Nature* **469**, 407–410 (2011).

12. Vaney, D. I., Sivyer, B. & Taylor, W. R. Direction selectivity in the retina: symmetry and asymmetry in structure and function. *Nat. Rev. Neurosci.* **13**, 194–208 (2012).

13. Helmstaedter, M. *et al.* Connectomic reconstruction of the inner plexiform layer in the mouse retina. *Nature* **500**, 168–174 (2013).

14. Jain, V. *et al.* Gain control by sparse, ultra-slow glycinergic synapses. *Cell Rep.* **38**, 110410 (2022).

15. Haverkamp, S. & Wässle, H. Characterization of an Amacrine Cell Type of the Mammalian Retina Immunoreactive for Vesicular Glutamate Transporter 3. *J. Comp. Neurol.* **468**, 251–263 (2004).

16. Kim, T., Soto, F. & Kerschensteiner, D. An excitatory amacrine cell detects object motion and provides feature-selective input to ganglion cells in the mouse retina. *Elife* **4**, 1–13 (2015).

17. Della Santina, L. *et al.* Glutamatergic Monopolar Interneurons Provide a Novel Pathway of Excitation in the Mouse Retina. *Curr. Biol.* **26**, 2070–2077 (2016).

18. Lee, S. *et al.* An unconventional glutamatergic circuit in the retina formed by

vGluT3 amacrine cells. *Neuron* **84**, 708–715 (2014).

19. Krishnaswamy, A., Yamagata, M., Duan, X., Hong, Y. K. & Sanes, J. R. Sidekick 2 directs formation of a retinal circuit that detects differential motion. *Nature* **524**, 466–470 (2015).

20. Lee, S., Zhang, Y., Chen, M. & Zhou, Z. J. Segregated Glycine-Glutamate Co-transmission from vGluT3 Amacrine Cells to Contrast-Suppressed and Contrast-Enhanced Retinal Circuits. *Neuron* **90**, 27–34 (2016).

21. Tien, N. W., Kim, T. & Kerschensteiner, D. Target-Specific Glycinergic Transmission from VGluT3-Expressing Amacrine Cells Shapes Suppressive Contrast Responses in the Retina. *Cell Rep.* **15**, 1369–1375 (2016).

22. Mani, A. & Schwartz, G. W. Circuit Mechanisms of a Retinal Ganglion Cell with Stimulus-Dependent Response Latency and Activation Beyond Its Dendrites. *Curr. Biol.* **27**, 471–482 (2017).

23. Lee, S., Chen, M., Shi, Y. & Zhou, Z. J. Selective glycinergic input from vGluT3 amacrine cells confers a suppressed-by-contrast trigger feature in a subtype of M1 ipRGCs in the mouse retina. *J. Physiol.* **0**, 1–14 (2021).

24. Liu, J. & Sanes, J. R. Cellular and molecular analysis of dendritic morphogenesis in a retinal cell type that senses color contrast and ventral motion. *J. Neurosci.* **37**, 12247–12262 (2017).

25. Rousso, D. L. *et al.* Two Pairs of ON and OFF Retinal Ganglion Cells Are Defined by Intersectional Patterns of Transcription Factor Expression. *Cell Rep.* **15**, 1930–1944 (2016).

26. Jacoby, J. & Schwartz, G. W. Three small-receptive-field ganglion cells in the

mouse retina are distinctly tuned to size, speed, and object motion. *J. Neurosci.* **37**, 610–625 (2017).

27. Schmidt, T. M. & Kofuji, P. Functional and Morphological Differences among Intrinsically Photosensitive Retinal Ganglion Cells. *J. Neurosci.* **29**, 476 LP – 482 (2009).

28. Stabio, M. E. *et al.* The M5 Cell: A Color-Opponent Intrinsically Photosensitive Retinal Ganglion Cell. *Neuron* **97**, 150-163.e4 (2018).

29. Johnson, K. P., Zhao, L. & Kerschensteiner, D. A Pixel-Encoder Retinal Ganglion Cell with Spatially Offset Excitatory and Inhibitory Receptive Fields. *Cell Rep.* **22**, 1462–1472 (2018).

30. Jia, Y., Lee, S., Zhuo, Y. & Jimmy Zhou, Z. A retinal circuit for the suppressed-by-contrast receptive field of a polyaxonal amacrine cell. *Proc. Natl. Acad. Sci. U. S. A.* **117**, 9577–9583 (2020).

31. Grimes, W. N., Seal, R. P., Oesch, N., Edwards, R. H. & Diamond, J. S. Genetic targeting and physiological features of VGLUT3+ amacrine cells. *Vis. Neurosci.* **28**, 381–392 (2011).

32. Grimes, W. N. *et al.* A high-density narrow-field inhibitory retinal interneuron with direct coupling to Müller glia. *J. Neurosci.* JN-RM-0199-20 (2020). doi:10.1101/2020.01.23.917096

33. Wang, J. *et al.* Anatomy and spatial organization of Müller glia in mouse retina. *J. Comp. Neurol.* **525**, 1759–1777 (2017).

34. Bae, J. A. *et al.* Digital Museum of Retinal Ganglion Cells with Dense Anatomy and Physiology. *Cell* **173**, 1293-1306.e19 (2018).

35. Grimes, W. N., Zhang, J., Graydon, C. W., Kachar, B. & Diamond, J. S. Retinal Parallel Processors: More than 100 Independent Microcircuits Operate within a Single Interneuron. *Neuron* **65**, 873–885 (2010).

36. Zhu, Y., Xu, J., Hauswirth, W. W. & DeVries, S. H. Genetically targeted binary labeling of retinal neurons. *J. Neurosci.* **34**, 7845–7861 (2014).

37. Jacoby, J., Zhu, Y., DeVries, S. H. & Schwartz, G. W. An Amacrine Cell Circuit for Signaling Steady Illumination in the Retina. *Cell Rep.* **13**, 2663–2670 (2015).

38. Park, S. J. H. *et al.* Convergence and divergence of CRH amacrine cells in mouse retinal circuitry. *J. Neurosci.* **38**, 3753–3766 (2018).

39. Sabbah, S., Berg, D., Papendorp, C., Briggman, K. L. & Berson, D. M. A cre mouse line for probing irradiance- and direction-encoding retinal networks. *eNeuro* **4**, 1–21 (2017).

40. Jacoby, J., Nath, A., Jessen, Z. F. & Schwartz, G. W. A Self-Regulating Gap Junction Network of Amacrine Cells Controls Nitric Oxide Release in the Retina. *Neuron* **100**, 1149-1162.e5 (2018).

41. Kim, T. & Kerschensteiner, D. Inhibitory Control of Feature Selectivity in an Object Motion Sensitive Circuit of the Retina. *Cell Rep.* **19**, 1343–1350 (2017).

42. Della Santina, L. *et al.* Glutamatergic Monopolar Interneurons Provide a Novel Pathway of Excitation in the Mouse Retina. *Curr. Biol.* **26**, 2070–2077 (2016).
