# Supplementary material for: A circuit suppressing retinal drive to the optokinetic system during fast image motion
Source: Nat Commun. 2023 Aug 23;14:5142. doi: 10.1038/s41467-023-40527-z (PMC10447436; doi:10.1038/s41467-023-40527-z)
Supplement: Supplementary file 3 — Reporting Summary [file 41467_2023_40527_MOESM3_ESM.pdf]

nature portfolio

Corresponding author(s): David Berson

Last updated by author(s): May 20, 2023

Reporting Summary

Nature Portfolio wishes to improve the reproducibility of the work that we publish. This form provides structure for consistency and transparency in reporting. For further information on Nature Portfolio policies, see our Editorial Policies and the Editorial Policy Checklist.

Statistics

For all statistical analyses, confirm that the following items are present in the figure legend, table legend, main text, or Methods section.

n/a	Confirmed	
☐	☒	The exact sample size (*n*) for each experimental group/condition, given as a discrete number and unit of measurement
☐	☒	A statement on whether measurements were taken from distinct samples or whether the same sample was measured repeatedly
☐	☒	The statistical test(s) used AND whether they are one- or two-sided<br>*Only common tests should be described solely by name; describe more complex techniques in the Methods section.*
☒	☐	A description of all covariates tested
☒	☐	A description of any assumptions or corrections, such as tests of normality and adjustment for multiple comparisons
☐	☒	A full description of the statistical parameters including central tendency (e.g. means) or other basic estimates (e.g. regression coefficient) AND variation (e.g. standard deviation) or associated estimates of uncertainty (e.g. confidence intervals)
☐	☒	For null hypothesis testing, the test statistic (e.g. *F*, *t*, *r*) with confidence intervals, effect sizes, degrees of freedom and *P* value noted<br>*Give P values as exact values whenever suitable.*
☒	☐	For Bayesian analysis, information on the choice of priors and Markov chain Monte Carlo settings
☒	☐	For hierarchical and complex designs, identification of the appropriate level for tests and full reporting of outcomes
☒	☐	Estimates of effect sizes (e.g. Cohen's *d*, Pearson's *r*), indicating how they were calculated

Our web collection on statistics for biologists contains articles on many of the points above.

Software and code

Policy information about availability of computer code

Data collection	Fluoview 4.1 (Olympus), MultiClamp Commander 700B, Clampex 10.4, Matlab R2018a custom written routines using the Psychophysics toolbox.
Data analysis	Matlab R2015a custom written routines, WebKnossos (https://webknossos.org/) v. 0.12.3. Fiji version 2.1.0/1.53f.

For manuscripts utilizing custom algorithms or software that are central to the research but not yet described in published literature, software must be made available to editors and reviewers. We strongly encourage code deposition in a community repository (e.g. GitHub). See the Nature Portfolio guidelines for submitting code & software for further information.

Data

Policy information about availability of data

All manuscripts must include a data availability statement. This statement should provide the following information, where applicable:

- Accession codes, unique identifiers, or web links for publicly available datasets
- A description of any restrictions on data availability
- For clinical datasets or third party data, please ensure that the statement adheres to our policy

Data Availability
Source data are provided with this paper. Single cell recordings and calcium imaging data are available on https://doi.org/10.5281/zenodo.794713553. Electron-microscopic reconstructions

are available on knossos.org/links/EBfAuezVRmNWiAt4.

Human research participants

Policy information about studies involving human research participants and Sex and Gender in Research.

Reporting on sex and gender	N\A
Population characteristics	N\A
Recruitment	N\A
Ethics oversight	N\A

Note that full information on the approval of the study protocol must also be provided in the manuscript.

Field-specific reporting

Please select the one below that is the best fit for your research. If you are not sure, read the appropriate sections before making your selection.

[x] Life sciences   [ ] Behavioural & social sciences   [ ] Ecological, evolutionary & environmental sciences

For a reference copy of the document with all sections, see nature.com/documents/nr-reporting-summary-flat.pdf

Life sciences study design

All studies must disclose on these points even when the disclosure is negative.

Sample size	No sample size calculation was performed. Due to the difficulty and low throughput of the experiments, a small sample (~3-5 repetitions) was collected that allowed us to see clear effects if these existed. If time allowed, we added more data to improve the statistical strength of the results.
Data exclusions	Patch-clamp measurements were excluded if cells seemed unhealthy or the quality of the patch was low. Some optogenetics measurements were excluded due to phenomena that had to do with incomplete phototransduction blockade, see text. ROIs in calcium imaging were excluded if they had a very different or very noisy response compared to the rest of the ROIs, or if ROIs were marked on ganglion cell dendrites and not VGluT3.
Replication	Information regarding the numbers of independent experiments is included under "Statistics and Reproducibility" in the main text. Dates of the experiments appear in the Source Data file. The variance in the results is detailed in the figures, and considerations for omitting data were included in Methods and in "Data exclusions" above.
Randomization	In all stimuli where one parameter was varied (e.g. speed, direction, spot size), presentations with different parameter values were used in a random order.
Blinding	Blinding is irrelevant and impractical in these experiments. A cell-type needs to be identified and stimulus/pharmacology conditions have to be carefully controlled during the experiment by the experimenter, while also looking at the acquired data in real-time to identify potential problems. Therefore hiding any of these elements from the experimenter would be difficult.

Reporting for specific materials, systems and methods

We require information from authors about some types of materials, experimental systems and methods used in many studies. Here, indicate whether each material, system or method listed is relevant to your study. If you are not sure if a list item applies to your research, read the appropriate section before selecting a response.

Materials & experimental systems

n/a	Involved in the study	
[ ]	[x]	Antibodies
[x]	[ ]	Eukaryotic cell lines
[x]	[ ]	Palaeontology and archaeology
[ ]	[x]	Animals and other organisms
[x]	[ ]	Clinical data
[x]	[ ]	Dual use research of concern

Methods

n/a	Involved in the study	
[x]	[ ]	ChIP-seq
[x]	[ ]	Flow cytometry
[x]	[ ]	MRI-based neuroimaging

Antibodies

Antibodies used	Primary antibodies: Goat anti-ChAT (Choline acetyltransferase; 1:200, Millipore Sigma #AB144); Rabbit anti-VGluT3 (1:250, Invitrogen #PA5-85784). Chicken anti-GFP (1:1000, Abcam #ab13970) was used to enhance the fluorescence of the Cre-dependent GFP virus. Rabbit anti-HA tag (1: 200, Cell Signaling Technology #3724) was used to stain the HA-tagged hM4Di receptor in the VGluT3 x DREADD mouse. Secondary antibodies: Donkey anti-Chicken 488 (1:1000, Jackson Immunoresearch #703-545-155); Donkey anti-Chicken 594 (1:1000, Jackson Immunoresearch #703-585-155); Donkey anti-Goat 488 (1:200, Invitrogen #A-11055); Donkey anti-Goat 594 (1:200, Invitrogen #A-11058); Donkey anti-Rabbit 647 (1:200, Invitrogen #A-31573).
Validation	We did not validate our antibodies. We used commercially available antibodies designated for use in the desired host species by the manufacturers. Testing results or references for previous published work using the antibodies are provided by the manufacturers, in the web pages below:<br><br>Primary:<br>https://www.emdmillipore.com/US/en/product/Anti-Choline-Acetyltransferase-Antibody,MM_NF-AB144#documentation<br>https://www.thermofisher.com/antibody/product/VGLUT3-Antibody-Polyclonal/PA5-85784<br>https://www.scbt.com/p/gfp-antibody-c-2?gclid=CjwKCAjwvJyjBhApEiwAWz2nLdt1hOgyGiTpqxOWeOv69l7f2x9j96aNQX1JczaNZLqXtn2nkvnsdhoC-1YQAvD_BwE<br>https://www.cellsignal.com/products/primary-antibodies/ha-tag-c29f4-rabbit-mab/3724?gclid=CjwKCAjwvJyjBhApEiwAWz2nLQ30WMoXTGtNzao1wZJ6uW7ts5XhgFr4V_k80J-7EgabjBe2qwSn8hoCuVIQAvD_BwE&gclsrc=aw.ds<br><br>Secondary:<br>https://www.jacksonimmuno.com/catalog/products/703-545-155<br>https://www.jacksonimmuno.com/catalog/products/703-585-155<br>https://www.thermofisher.com/antibody/product/Donkey-anti-Goat-IgG-H-L-Cross-Adsorbed-Secondary-Antibody-Polyclonal/A-11055<br>https://www.thermofisher.com/antibody/product/Donkey-anti-Goat-IgG-H-L-Cross-Adsorbed-Secondary-Antibody-Polyclonal/A-11058<br>https://www.thermofisher.com/antibody/product/Donkey-anti-Goat-IgG-H-L-Cross-Adsorbed-Secondary-Antibody-Polyclonal/A-31573

Animals and other research organisms

Policy information about studies involving animals; ARRIVE guidelines recommended for reporting animal research, and Sex and Gender in Research

Laboratory animals	We studied adult mice of either sex, 2–8 months old. Wild-type mice were C57BL/6J (Jackson Laboratory). To target ON DSGCs for recording, we used HoxD10-GFP mice (GENSAT collection, Tg(Hoxd10-EGFP)LT174Gsat/Mmucd, MMRRC #032065) and Pcdh9-Cre mice (GENSAT collection, Tg(Pcdh9-Cre)NP276Gsat/Mmucd, MMRRC #036084). The VGluT3-Cre line (The Jackson Laboratory, B6;129S-Slc17a8tm1.1(cre)Hze/J, #028534) was crossed with each of four Cre-dependent mouse lines: Ai32 (Jackson, B6.Cg-Gt(ROSA)26Sortm32(CAG-COP4*H134R/EYFP)Hze/J, #024109) for optogenetics; DREADD mice (Jackson, B6.129-Gt(ROSA)26Sortm1(CAG-CHRM4*,-mCitrine)Ute/J, #026219) for chemogenetics, Ai14 (Jackson, B6;129S6-Gt(ROSA)26Sortm14(CAG-tdTomato)Hze/J, #007908) for fluorescent labeling for characterization of the VGluT3-Cre mouse, and Ai148 (Jackson, B6.Cg-Igs7tm148.1(tetO-GCaMP6f,CAG-tTA2)Hze/J, #030328) for Ca2+ imaging. For the Müller cell control experiment, GLAST-Cre mice (Jackson, Tg(Slc1a3-cre/ERT)1Nat/J, #012586) were crossed with Ai32.
Wild animals	No wild animals were used in the study
Reporting on sex	We studied mice of either sex. No sex based analysis was performed.
Field-collected samples	No field collected samples were used in the study.
Ethics oversight	All procedures were in accordance with the National Institutes of Health guidelines and approved by the Institutional Animal Care and Use Committee at Brown University.

Note that full information on the approval of the study protocol must also be provided in the manuscript.
